# Medicine and “Graft”

**Published:** 1904-06

**Authors:** 


### Medicine and "Graft."

THE Physician's Club, of Chicago, had a dinner recently at which the main subject of discussion was "Graft." Several eminent laymen of the Windy City were present and gave their definitions of graft in various intelligent manner. Mr. William H. McSurley defined a "grafter" as "one who attaches himself to some outside interest, some interest apart from the main motive of his life, for the purpose of getting from that outside interest something that will be beneficial to the main motive of his life." This is a clever definition and quite covers the ground from the standpoint of ethics.

Dr. Lydston mentioned the physician in politics and public affairs and said "the doctor who devotes a portion of his time to public affairs is forever damned in the eyes of the public and profession, while the reverse is true of the lawyer."

There are few lawyers who render public service without receiving pay for it. On the other hand, much is done by physicians who for years have labored for the public good, gaining nothing for their service save possibly the ill will of their fellow practitioners and the sneers of the well-served public. This has been illustrated frequently in recent years. For this condition of affairs the profession has itself to blame. There is a sad lack of unity. If a physician takes an active part in public affairs he is looked upon with suspicion; let him seek office and he is a "grafter;" let him raise his voice in any but a general way, except it be in accord with the cut and dried principles of professional utterance, and he is damned as a self-advertiser. The profession is broad-minded in a narrow way; it is encrusted with the tradition of musty ages.

A physician is by natural training a diplomat and is perforce politic in his dealings and, as a whole, the profession is politic; but there is a wide difference between being politic and political. The profession is too retiring; it is afraid of itself and as a consequence the members, who by right of education and training are fitted for the highest position in a community where public welfare should be considered paramount to political graft, sit back and are becoming the playthings of the practical politician who sees good only in graft, which in its properly understood, practical sense means getting something tangible, either money or position which brings money easily, without doing anything for it or giving an equivalent. It may be safely and truthfully said there is no graft among the legitimate practitioners of medicine.

